# Balance Control and Its Correlation with Functional Capacity in People with Post-Tuberculosis Lung Disease

**DOI:** 10.3390/muscles5030064

**Published:** 2026-09-16

**Authors:** Pedro Henrique Perpetuo de Lima Silva, Isabelle da Nobrega Ferreira, Alessandro dos Santos Beserra, Nielza Moreira de Souza, Estephane Ramos de Souza Penna, Alícia Sales Carneiro, Walter Costa, Bruna Cuoco Provenzano, Ana Paula Santos, Agnaldo José Lopes

**Affiliations:** 1 Rehabilitation Sciences Post-Graduation Program, Centro Universitário Augusto Motta (UNISUAM), Rua Dona Isabel, 94, Bonsucesso, Rio de Janeiro 21032-060, Brazil; pedroperpetuo@souunisuam.com.br (P.H.P.d.L.S.); nielzasouza@souunisuam.com.br (N.M.d.S.); 2 Postgraduate Program in Medical Sciences, School of Medical Sciences, Universidade do Estado do Rio de Janeiro (UERJ), Avenida Professor Manoel de Abreu, 444, 2° Andar, Vila Isabel, Rio de Janeiro 20550-170, Brazil; isabellenob@gmail.com (I.d.N.F.); alessandro.santos.br@gmail.com (A.d.S.B.); aliciasc6@gmail.com (A.S.C.); anapsantos.ip@gmail.com (A.P.S.); 3 Faculty of Physiotherapy, Centro Universitário Augusto Motta (UNISUAM), Avenida, Paris, 84, Bonsucesso, Rio de Janeiro 21041-020, Brazil; estephanepenn@gmail.com; 4 Department of Pulmonology, Hospital Universitário Pedro Ernesto, Universidade do Estado do Rio de Janeiro (UERJ), Boulevard 28 de Setembro, 77, Vila Isabel, Rio de Janeiro 20551-030, Brazil; dr.waltercosta@terra.com.br (W.C.); brunaclinicamedica@gmail.com (B.C.P.); 5 Medical School and Institute of Thorax Diseases, Universidade Federal do Rio de Janeiro (UFRJ), Rua Professor Rodolpho Paulo Rocco, 255, Sala 01D 58/60, Cidade Universitária, Rio de Janeiro 21941-913, Brazil

**Keywords:** tuberculosis, balance control, exercise, muscle strength, pulmonary function

## Abstract

Background: Because falls can lead to loss of autonomy in individuals with post-tuberculosis lung disease (iwPTLD), assessing fall risk is crucial for reducing the likelihood of fractures and preventing the decline of physical function. This cross-sectional study aimed to evaluate balance control (BC) in iwPTLD and correlate it with functional capacity during exertion (FCE), pulmonary function, muscle strength, and fatigue. Methods: This cross-sectional study assessed BC in iwPTLD using the Berg Balance Scale (BBS), the Tinetti Balance Scale (TBS), and the Timed “Up and Go” Test (TUGT). Participants also underwent assessments of pulmonary function, respiratory muscle strength, handgrip strength (HGS), quadricep strength (QMS), general fatigue, and functional capacity during exertion (FCE) using the six-minute step test (6MST). Results: Of the 50 individuals with iwPTLD, 66%, 38%, and 46% had a BBS, TBS, and TUGT, respectively, indicating a moderate/high risk of falls. The degree of agreement between BC classifications was weak to poor (Kappa between 0.15 and 0.35). The BBS and TBS exhibited weak-to-moderate correlations with the QMS and 6MST. The TUGT, however, showed strong correlations with the QMS, the 6MST, and residual volume/total lung capacity ratio. Conclusions: Impaired BC appears to be common in iwPTLD, with alterations observed in up to two-thirds of cases. For these individuals, there are significant correlations of BC with peripheral muscle strength, FCE, and static pulmonary hyperinflation, especially when the TUGT is used to assess BC. Thus, the TUGT may be a tool with potential clinical applications for identifying balance impairment in patients with PTLD. However, it requires prospective validation. The BBS and TBS should be reserved for comprehensive assessments of patients with abnormal TUGT results.

## 1. Introduction

Post-tuberculosis lung disease (PTLD) is a global health challenge that affects an estimated 155 million pulmonary tuberculosis (PTB) survivors following treatment [1]. While most individuals with PTB are cured during the acute phase of the disease, nearly half of the annual health impact of tuberculosis (TB)—58 million disability-adjusted life years—is attributable to post-TB complications [2]. PTLD is associated with chronic symptoms and persistent imaging abnormalities, such as residual pulmonary fibrosis, cavitation, and structural distortion, which ultimately lead to lung remodeling that affects respiratory function [3]. Consequently, PTLD can cause airflow obstruction, restrictive ventilatory disorders, and impaired gas exchange [4].

In addition to respiratory involvement, extrapulmonary manifestations of PTLD include muscle weakness, difficulty performing physical activities, and impaired mental health [5]. Following TB treatment, residual lung damage triggers physical dysfunction and reduced exercise capacity. This is often further impaired by musculoskeletal dysfunction in these patients [6]. In PTLD, physical inactivity, impaired skeletal muscle function, and difficulty performing activities of daily living are interrelated. These factors can lead to a progressive loss of physical function [7]. In fact, a recent study showed that handgrip strength (HGS) and quadricep muscle strength (QMS) were reduced in 34% and 25.5% of adults with PTLD, respectively [8]. Furthermore, the authors showed that alongside reduced diffusion capacity of the lungs for carbon monoxide (DLco), QMS and HGS explain approximately 40% of the variability in functional capacity during exertion (FCE) in this patient population. Thus, the interrelationship between peripheral muscle strength and low FCE indicates that systemic symptoms need to be routinely measured in individuals with PTLD (iwPTLD).

Several factors directly influence balance control (BC), including muscle strength, reaction time, joint flexibility, sensory integration, and various adaptive neuromotor strategies. BC depends on neuromuscular synergy and the integration of information from multiple subsystems, such as the vestibular and somatosensory systems [9]. In iwPTLD, limited thoracic expansion and altered respiratory mechanics can interfere with trunk mobility and center of mass control. Thus, anterior trunk tilt and semi-flexed posture can reduce the base of support and increase instability, leading to alterations in BC [10]. In addition to the respiratory system, central nervous system (CNS) involvement by TB (CNSTB) can lead to gait disturbances and impact BC. However, CNSTB is frequently overlooked and underdiagnosed in clinical contexts such as active PTB and PTLD. The diagnosis of CNSTB is difficult due to the disease’s rarity, nonspecific presentation, and low-sensitivity diagnostic tests. Subtle neuromuscular sequelae may persist in iwPTLD [11]. Damage to blood vessels, inflammation, and immunological alterations are some of the proposed mechanisms of CNS injury due to tuberculosis [12].

Individuals with pulmonary dysfunction, such as PTLD, often have muscular and ventilatory impairments that affect motor coordination and posture. Furthermore, alterations in gait pattern, compensatory body stiffness, and cognitive deficits related to chronic oxygen deprivation are common in individuals with chronic lung diseases. These factors compromise anticipatory postural control and reactive mechanisms, increasing the risk of falls [13,14]. Therefore, assessing balance control (BC) is essential to prevent accidents and guide physiotherapy interventions focused on improving overall function. It is important to note that alterations in BC may persist or worsen after PTLD treatment because many of these alterations are not diagnosed during the acute TB phase [12]. Moreover, musculoskeletal dysfunction can deteriorate physical function and impair BC in iwPTLD, similar to what occurs in chronic obstructive pulmonary disease (COPD). Thus, this study aimed to evaluate BC in iwPTLD and correlate it with FCE, pulmonary function, muscle strength, and fatigue.

## 2. Materials and Methods

### 2.1. Subjects and Ethics

From April 2024 to May 2025, a cross-sectional study of iwPTLD was conducted at the Hospital Universitário Pedro Ernesto of the Universidade do Estado do Rio de Janeiro, Brazil. These patients were consecutively recruited from the TB outpatient clinic at the hospital in question. They were recruited either immediately after completing anti-TB treatment at the TB outpatient clinic or during their follow-up at the PTLD outpatient clinic. Inclusion criteria: iwPTLD patients aged 18 years or older who had completed anti-TB therapy within the previous three years. Exclusion criteria: a prior diagnosis of a disease affecting the bones or peripheral muscles; a history of CNSTB; or cognitive limitations that prevent comprehension of recommendations. The study was conducted in accordance with the Declaration of Helsinki and approved by the Research Ethics Committee of the Hospital Universitário Pedro Ernesto of the Universidade do Estado do Rio de Janeiro (CAAE-70493823.5.0000.5259; 25 August 2023). Written informed consent was obtained from all participants.

### 2.2. Assessments

Three tools were used to evaluate the BC. First, participants were assessed using the Berg Balance Scale (BBS). The BBS evaluates functional balance performance through 14 tests examining an individual’s ability to sit, stand, reach for objects, turn their body, look over their shoulders, maintain a one-leg stance, and step onto a platform. The scale ranges from 0 to 56 points, with each test offering five scoring options from 0 to 4 points. Based on the BBS, which has been used with older adults and replicated with other populations, we defined a score below 46 as high risk, a score between 46 and 53 as moderate risk, and a score above 53 as low risk [15]. We adopted this interpretive strategy because there are no established cutoff points for iwPTLD. Thus, these scores for fall risk classification should not be interpreted as validated thresholds specific to PTLD. Next, the participants were evaluated using the Tinetti Balance Scale (TBS). The TBS evaluates aspects of gait, such as speed, step length, symmetry, and standing balance, as well as turning and changes performed with the eyes closed. A score below 19 on the TBS indicates a high risk of falls. A score between 19 and 24 indicates moderate risk, and a score above 24 (up to a maximum of 28) indicates low risk [16]. However, it is important to note that these scores should not be interpreted as a validated fall risk threshold specific to PTLD, as they have only been validated for patients with COPD. Finally, the participant took the Timed “Up and Go” Test (TUGT). Patients sit on a standard 45 cm high chair with their backs against the backrest. They are then instructed to stand up and walk as quickly and safely as possible along the floor for three meters in a straight line, and then return to the chair and sit down in the starting position. Since there are no specific cutoff values for PTLD, the TUGT results were interpreted based on a study of elderly individuals at risk of falling [17]. The results were categorized as follows: high risk of falls (>20 s), moderate risk of falls (10–20 s), or low risk of falls (<10 s) [17]. The value of 8.42 s in the TUGT has been previously validated as a cutoff point for normal physical capacity in patients with COPD [18]. We used this cutoff point because there are no validated cutoff values specifically for the PTLD population. However, iwPTLD are at an increased risk for and have a high prevalence of COPD [19].

We assessed pulmonary function using spirometry and body plethysmography to measure static lung volumes. We also assessed DLco and respiratory muscle strength. All pulmonary function tests were performed using the HDpft 3000 device (nSpire Health, Inc., Longmont, CO, USA). National predicted equations were used to interpret the participants’ pulmonary function [20,21,22,23].

An isometric dynamometer (SH5001, Saehan Corporation, Seoul, Republic of Korea) was used to assess HGS. The highest value was recorded when participants took three measurements, with at least one minute between each one. Cutoff points of 16 and 27 kgf were used for women and men, respectively [24]. QMS was assessed using a traction dynamometer (model E-lastic 5.0, E-sporte SE, Brasília, Brazil) on the dominant leg. The highest value among three 5 s sustained contractions with one-minute intervals between them was selected for analysis. The cutoff points used were 14.8 and 25.3 kgf for women and men, respectively [25].

We used the Functional Assessment of Chronic Illness Therapy-Fatigue (FACIT-F) scale to measure fatigue. The scale has 13 questions, and scores range from 0 to 52. A higher score indicates lower fatigue. A cutoff point of ≤34 indicates fatigue [26].

The FCE was assessed using the six-minute step test (6MST). This test was performed using a 20 cm high, 80 cm long, 40 cm wide wooden step with a non-slip surface. Volunteers were instructed to step up and down at a pace that would allow them to take the greatest number of steps possible within six minutes. They could alternate their lower limbs during the stepping movements, but their upper limbs had to remain stationary at their sides. To make comparisons with the measurements obtained from the participants, national reference equations were used [27].

### 2.3. Statistical Analysis

Using MedCalc^®^ software (MedCalc Software, Mariakerke, Belgium, version 11.0.1), we estimated the sample size required to conduct the study. Since the main analysis centered on the TUGT as the primary outcome and its primary association with the 6MST, an expected Spearman correlation coefficient (*r_s_*) of 0.5 was adopted with a 5% significance level and 80% statistical power (1 − *β* = 0.80). Based on these parameters, the minimum estimated sample size was 29 participants [28].

The Shapiro–Wilk test was used to determine if each variable in the study followed a Gaussian distribution. Considering that the scale scores did not present a normal distribution, the correlation between BC assessments and numerical variables was assessed using Spearman’s correlation coefficient. Missing data from body plethysmography (five participants) was addressed through pairwise exclusion in correlation analyses [29]. We performed a Mann–Whitney test to compare BC assessments according to categorical variables. The degree of agreement between BC assessment classifications was analyzed using the linear weighted Kappa coefficient according to Cohen. We used the linearly weighted Kappa coefficient based on the principle that the three classifications used to assess BC—low, moderate, and high risk of falling—have intuitive meanings, with each classification representing a different degree of practical deviation [30]. The following cut-off points were used for the interpretation of Kappa: ≤0.20 = poor agreement, 0.21–0.40 = weak agreement, 0.41–0.60 = moderate agreement, 0.61–0.80 = good agreement, and >0.80 = excellent agreement [30]. The significance level adopted was 5%. Statistical analyses were performed using IBM SPSS Statistics version 26.0 (IBM Corp., Armonk, NY, USA).

## 3. Results

### 3.1. Patient Characteristics

Of the 53 patients evaluated for inclusion in the study, three were excluded due to neurological and/or musculoskeletal diseases unrelated to TB. No one refused to participate in the study. Five patients were unable to undergo body plethysmography: three because they had difficulty performing rapid, shallow breathing cycles, and two because they were claustrophobic.

None of the participants had drug-resistant PTB. Thirty-four participants (68%) were women with a mean age of 50.8 ± 15.4 years. The average body mass index was 25.2 ± 6.7 kg/m^2^, and the median time since the end of PTB treatment was 24 (18–30) months. Thirteen (26%) of the iwPTLD had a history of smoking, with an average smoking load of 14 (6–23) pack-years.

### 3.2. Assessment of Balance Control

Upon evaluating the BC, we found that the median BBS score was 52 (46–56). Thirty-three (66%) iwPTLD had a BBS score of ≤53, indicating a moderate/high risk of falls. In the TBS, the median score was 26 (22–27), and 19 (38%) participants had a score of ≤24, indicating a moderate/high risk of falls. In the TUGT, the median was 9.3 (8.2–11), and 23 (46%) participants took ≥10 s to complete the test, indicating a moderate/high risk of falls. Considering the cutoff point of 8.42 s used for normal physical capacity in COPD patients, only 11 (22%) participants had good performance. Table 1 shows the anthropometric data, BC, and functionality of the participants.

### 3.3. Agreement Between Balance Control Assessments

We observed weak, yet statistically significant, agreement between the BBS and TBS classifications when measuring the degrees of agreement between the BC classifications (Kappa = 0.35; *p* = 0.001). Weak but statistically significant agreement was also found between TBS and TUGT (Kappa = 0.25, *p* = 0.014). However, the comparison between the BBS and the TUGT showed poor agreement (Kappa = 0.15, *p* = 0.085). Table 2 shows the degree of agreement between the BC assessments using the percentage of agreement, the linear weighted Kappa coefficient, and the 95% confidence interval of Kappa.

### 3.4. Pulmonary Function, Muscle Function, and Functional Capacity During Exertion

Of the participants who underwent pulmonary function testing, only 14 (28%) had normal results. However, 14 (28%), 13 (26%), and nine (18%) participants exhibited obstructive, restrictive, and mixed patterns, respectively. Thirty-one (62%) participants had elevated airway resistance, and 22 (44%) had reduced DLco. In the muscle function assessment, 38 (78%) and 30 (60%) iwPTLD exhibited reduced maximum expiratory pressure and maximum inspiratory pressure (MIP), respectively. HGS and QMS were reduced in 8% and 20% of participants, respectively. In the sample, 21 (42%) iwPTLD had a FACIT-F scale of ≤34, indicating fatigue. The median number of steps climbed in the 6MST was 83 (49–101), with 43 (86%) participants obtaining values <80% of predicted for the Brazilian population. Data on pulmonary function, muscle function, and FCE are shown in Table 3.

### 3.5. Correlations Between Balance Control Measures and Clinical Characteristics, Pulmonary Function, Muscle Function, and Functional Capacity During Exertion

**Figure 1:**
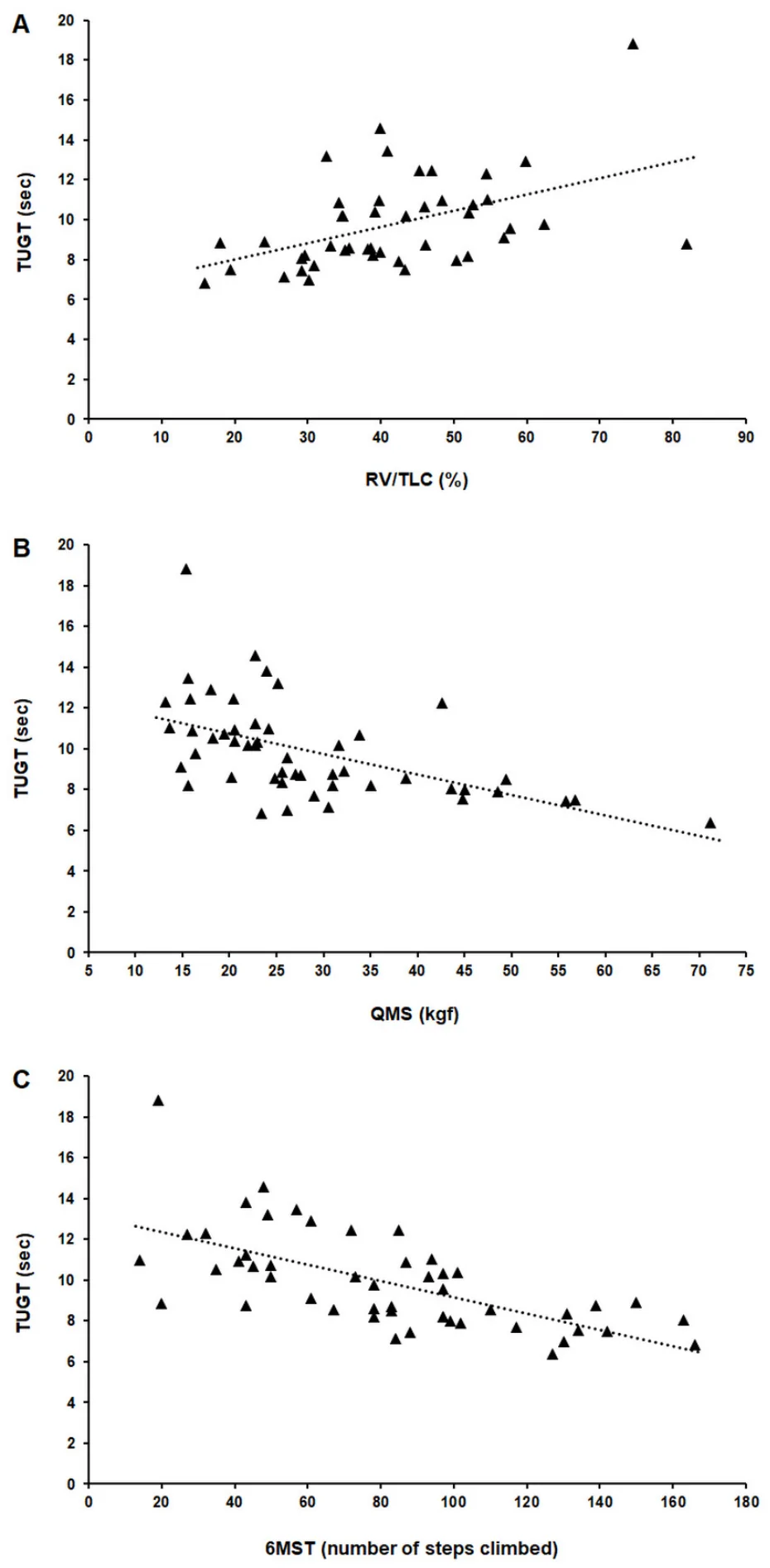
Relationship between Timed “Up and Go” Test (TUGT) and residual volume/total lung capacity (RV/TLC, *r_s_* = 0.500, *p* < 0.001) (**A**), quadricep muscle strength (QMS, *r_s_* = −0.641, *p* < 0.001) (**B**), and six-minute step test (6MST, *r_s_* = −0.696, *p* < 0.001) (**C**).

Finally, we examined the relationship between BC measures (BBS, TBS, and TUGT) and clinical characteristics, pulmonary function, muscle function, and FCE (Table 4 and Figure 1). BBS correlated significantly with age (*r_s_* = −0.362, *p* = 0.009), QMS (*r_s_* = 0.373, *p* = 0.007), and 6MST (*r_s_* = 0.290, *p* = 0.041). The TBS score was significantly correlated with the FACIT-F scale (*r_s_* = 0.376, *p* = 0.016), HGS (*r_s_* = 0.325, *p* = 0.021), MIP (*r_s_* = 0.293, *p* = 0.039), QMS (*r_s_* = 0.291, *p* = 0.040), and 6MST (*r_s_* = 0.310, *p* = 0.028). TUGT significantly correlated with smoking load (*r_s_* = 0.620, *p* = 0.023), the FACIT-F scale (*r_s_* = −0.340, *p* = 0.015), residual volume (RV)/total lung capacity ratio (TLC, *r_s_* = 0.500, *p* < 0.001), MIP (*r_s_* = −0.285, *p* = 0.044), HGS (*r_s_* = −0.335, *p* = 0.017), QMS (*r_s_* = −0.641, *p* < 0.001), and 6MST (*r_s_* = −0.696, *p* < 0.001).

## 4. Discussion

A better understanding of common PTLD phenotypes will help guide optimal treatment and allow for the identification of patient subgroups with distinct clinical characteristics and “treatable traits.” Therefore, assessing BC using validated functional scales enables the early detection of postural deficits, the prevention of falls, and the development of physiotherapy treatment plans. The main finding of this study is that up to two-thirds of iwPTLD have increased fall-risk classification. In these individuals, there is generally poor agreement between BC tests, suggesting that more than one type of test may be necessary for adequate assessment. Nearly all of these individuals have a low FCE, as assessed by the 6MST, which is correlated with poorer balance performance. In iwPTLD, lower QMS indicates worse BC. Furthermore, fatigue, static pulmonary hyperinflation (SPH), and reduced respiratory muscle strength correlate with impaired BC, particularly with the TUGT time. To the best of our knowledge, this is the first study to examine BC in iwPTLD.

The weak to poor agreement observed between the three balance assessment tools (weighted Kappa ranging from 0.15 to 0.35) indicates that they evaluate distinct domains of BC and should not be considered interchangeable. BBS predominantly assesses anticipatory postural control during static and quasi-dynamic tasks, TBS combines static balance with qualitative aspects of gait, whereas TUGT is an objective, time-based measure of functional mobility that integrates dynamic balance, lower limb power, and gait speed [15,16,17]. This distinction is further supported by our correlation findings: TUGT showed the strongest associations with QMS (*r_s_* = −0.641, *p* < 0.001) and functional capacity during exertion assessed by the 6MST (*r_s_* = −0.696, *p* < 0.001), while BBS correlated only weakly with 6MST (*r_s_* = 0.290, *p* = 0.041). Similar poor agreement between BBS and TUGT has been reported in COPD and post-COVID-19 populations, where TUGT was more sensitive to muscle dysfunction than BBS [15,31,32].

From a clinical perspective, we believe the TUGT is a promising tool for the prospective evaluation of iwPTLD. It is feasible, requiring less than two minutes, a chair, and a stopwatch. No specialized training is necessary. The TUGT is objective and strongly correlates with clinically relevant outcomes, such as static lung hyperinflation (RV/TLC), peripheral muscle weakness, and FCE. In our cohort, a cutoff of ≥10 s identified 46% of patients as being at moderate-to-high risk of falling. Only 22% of patients met the previously validated threshold for normal physical capacity in COPD patients (8.42 s) [18]. Pending future validation studies, patients with abnormal TUGT performance or a history of falls could undergo a more comprehensive assessment using the BBS to identify specific postural deficits and guide targeted physical therapy interventions. The TBS is valuable but may be less suitable as a standalone initial assessment tool until validation studies in PTLD are available due to its longer administration time and greater inter-rater variability in gait quality scoring [33,34].

In our sample, nearly half (46%) of iwPTLD exhibited some degree of airway obstruction, either in isolation or as part of a mixed ventilatory disorder. The pathophysiology of functional sequelae of PTB in PTLD is likely heterogeneous. It involves structural lung changes resulting from aberrant tissue repair and poorly understood mechanisms leading to airflow obstruction [4]. These mechanisms include smoking and the development of COPD [4]. Interestingly, we observed strong correlations between the TUGT time and both smoking load and SPH (increased RV/TLC). This points to the importance of airflow obstruction and chest wall derangement in the genesis of poor chest wall function in this population [10]. In PTLD, chest wall rigidity can limit the range of motion necessary for rapid postural adjustments. Dyspnea can also lead to inadequate postural strategies, such as forward trunk tilt and semi-flexed posture. These strategies reduce the base of support and increase instability [10]. Thus, rehabilitative strategies that prevent the deterioration of pulmonary function are essential in iwPTLD. These strategies can potentially minimize postural instability and BC in this population.

Peripheral muscle strength, particularly QMS, has been linked to limitations in heart rate, dyspnea, and the risk of hospitalization [35]. Similar to de Souza and colleagues [8], we found that approximately 20% of our sample had reduced QMS. Significant correlations were observed between QMS and all BC assessments, particularly with the TUGT (*r_s_* = −0.641, *p* < 0.001). These results highlight the importance of incorporating motor physiotherapy into a comprehensive rehabilitation program for iwPTLD. In iwPTLD, impaired muscle function negatively affects quality of life and has socioeconomic implications when it compromises the ability to perform work activities [36]. In agreement with the study by de Souza and colleagues [8], we also observed reduced HGS in a portion of our sample. In clinical practice, this finding is important because HGS assessment has proven to be an efficient, easily applicable method for estimating overall muscle strength and for early detection of sarcopenia in populations with chronic respiratory diseases [35]. Similar to QMS, the moderate correlation between reduced HGS and poor performance on the TUGT reinforces the importance of routinely assessing peripheral muscle strength in iwPTLD to improve BC in this population. It is also worth noting that nearly half of our iwPTLD experienced general fatigue, which was associated with poor BC. A better understanding of the factors associated with PTLD-related fatigue may elucidate new approaches to managing it, thus offering relief to a greater number of patients.

The 6MST is increasingly being used in clinical practice to assess FCE due to its low cost and strong association with various field tests, such as the 6 min walk test (6MWT) [27]. In our sample, performance during the 6MST was low, with only 14% of iwPTLD reaching values >80% of those predicted for the Brazilian population. While the healthy Brazilian population climbed an average of 175 ± 45 steps in the 6MST, the median in our sample was only 83 (49–101) steps. This value is very close to that observed in COPD patients, who climbed an average of 74 ± 29 steps [37]. Importantly, we found that performance in the 6MST significantly correlated with the three balance assessment tools used in our study, particularly the TUGT. These results suggest an association between poorer 6MST performance, postural instability and impaired BC in iwPTLD. It is worth noting that climbing stairs involves high-intensity aerobic physical exercise that requires muscular and cardiovascular effort. This leads to distinct physiological responses from those observed in the 6MWT and makes the metabolic and ventilatory demands more intense [38].

This study has limitations. Its cross-sectional design precludes causal inference. We did not perform neuroimaging; thus, subclinical CNSTB (tuberculomas, vasculitis) could not be excluded despite exclusion of clinically diagnosed CNSTB, and may have contributed to balance impairment [28]. We also lacked lung function and imaging data from the time of active PTB diagnosis, so the onset and trajectory of ventilatory and balance deficits could not be established. We used balance cutoffs validated in elderly and COPD populations, as no PTLD-specific thresholds exist. In particular, the COPD-derived TUGT threshold of 8.42 s should not be interpreted as a validated PTLD-specific fall-risk threshold. Several variables potentially associated with balance and functional performance, specifically age, sex, smoking exposure, and anthropometric characteristics, could act as confounders; however, the sample size prevented us from conducting reliable multivariate regression analyses for the primary balance outcomes. The statistical analyses performed in our study should be explicitly identified as exploratory: the interpretation of isolated *p*-values should be approached with caution given the significant risk of Type I error when performing multiple correlations. Finally, although we agree that BC may differ by ventilatory pattern, our sample size was insufficient for a robust subgroup analysis (mixed pattern *n* = 9); larger studies are needed to explore this hypothesis.

## 5. Conclusions

Impaired BC appears to be common in iwPTLD, with alterations observed in up to two-thirds of cases using the BC tests employed in routine practice. For these individuals, there are strong correlations between BC, as assessed by the TUGT, and SPH, QMS, and FCE. Weak-to-moderate correlations are found between BC, as measured by the BBS or TBS, and QMS and FCE. Thus, the TUGT may represent a tool with potential clinical application—requiring prospective validation—for identifying balance impairment in patients with PTLD, while reserving the BBS and TBS for comprehensive assessments of those with abnormal TUGT results. Although our findings are cross-sectional and hypothesis-generating in nature, they may serve as a starting point for randomized controlled trials evaluating BC in this population, particularly following physical reconditioning programs.

## Figures and Tables

**Table 1:** Anthropometry data, comorbidities, and balance control in the sample (*n* = 50).

Variables		Values
Sex (female/male)		34/16
Age (years), mean ± SD		50.8 ± 15.4
BMI (kg/m^2^), mean ± SD		25.2 ± 6.7
Comorbidities, number (frequency)	Hypertension	18 (36%)
	Diabetes	6 (12%)
	Asthma	3 (6%)
BBS, number (frequency)	Low risk of falling	17 (34%)
	Moderate risk of falling	22 (44%)
	High risk of falling	11 (22%)
TBS, number (frequency)	Low risk of falling	31 (62%)
	Moderate risk of falling	15 (30%)
	High risk of falling	4 (8%)
TUGT, number (frequency)	Low risk of falling	27 (54%)
	Moderate risk of falling	23 (46%)
	High risk of falling	0 (0%)

The values shown are the mean ± SD or number (frequency). BMI, body mass index; BBS, Berg Balance Scale; TBS, Tinetti Balance Scale; TUGT, Timed “Up and Go” Test.

**Table 2:** Degree of agreement between the classifications used to assess balance control in the sample (*n* = 50).

Comparisons	% Agreement	Weighted Kappa	95% CI Kappa	*p*-Value
BBS and TBS	60	0.35	0.16–0.52	0.001
BBS and TUGT	42	0.15	−0.02–0.32	0.085
TBS and TUGT	62	0.25	0.05–0.43	0.014

CI, confidence interval; BBS, Berg Balance Scale; TBS, Tinetti Balance Scale; TUGT, Timed “Up and Go” Test.

**Table 3:** Pulmonary function, muscle function, and functional capacity during exertion in the sample (*n* = 50).

Variables		Values
Pulmonary function	FVC (% predicted), mean ± SD	76 ± 24.1
	FEV_1_ (% predicted), mean ± SD	69.3 ± 26.6
	FEV_1_/FVC ratio (%), mean ± SD	74 ± 15.1
	FEF_25–75%_ (% predicted), mean ± SD	62.2 ± 39.7
	TLC (% predicted), mean ± SD *	86.5 ± 18.9
	RV (% predicted), mean ± SD *	103.4 ± 44.7
	RV/TLC ratio (%), mean ± SD *	41.8 ± 13.7
	Raw (cm H_2_O/L/s), median (interquartile range) *	4 (2–11)
	DLco (% predicted), mean ± SD	94.5 ± 34.9
Muscle function	MIP (% predicted), median (interquartile range)	47 (37–73)
	MEP (% predicted), median (interquartile range)	38 (26–56)
	HGS (kgf), median (interquartile range)	26 (20–31)
	QMS (kgf), median (interquartile range)	25 (20–33)
	FACIT-F scale (points), mean ± SD	34.3 ± 11.5
Functional capacity during exertion	6MST (number of steps climbed), median (interquartile range)	83 (49–101)
	6MST (% predicted), median (interquartile range)	50 (34–74)

The values shown are the mean ± SD or median (interquartile range). FVC, forced vital capacity; FEV_1_, forced expiratory volume in 1 s; FEF_25–75%_, forced expiratory flow during the middle half of the FVC maneuver; TLC, total lung capacity; RV, residual volume; Raw, airway resistance; DLco, diffusion capacity of the lungs for carbon monoxide; MIP, maximum inspiratory pressure; MEP, maximum expiratory pressure; HGS, handgrip strength; QMS, quadricep muscle strength; FACIT-F, Functional Assessment of Chronic Illness Therapy-Fatigue; 6MST, six-minute step test. * *n* = 45.

**Table 4:** Spearman’s correlation coefficients between postural balance measures, clinical characteristics, pulmonary function, muscle function, and functional capacity during exertion in people with post-tuberculosis lung disease (*n* = 50).

	Balance Control					
	BBS (Points)		TBS (Points)		TUGT (s)	
	*r_s_*	*p*-Value	*r_s_*	*p*-Value	*r_s_*	*p*-Value
Age (years)	**−0.362**	**0.009**	−0.220	0.12	0.272	0.056
BMI (kg/m^2^)	−0.118	0.41	0.064	0.66	−0.034	0.812
Time since the end of treatment (months)	−0.271	0.057	−0.053	0.72	0.147	0.315
Smoking load (pack-years)	−0.403	0.17	−0.080	0.79	**0.620**	**0.023**
FVC (% predicted)	0.169	0.24	0.097	0.50	−0.131	0.364
FEV_1_ (% predicted)	0.136	0.35	0.028	0.85	−0.166	0.257
FEV_1_/FVC ratio (%)	−0.091	0.53	−0.180	0.21	−0.180	0.218
FEF_25–75%_ (% predicted)	0.027	0.85	−0.128	0.37	−0.226	0.110
TLC (% predicted) *	0.087	0.58	0.065	0.68	0.029	0.860
RV (% predicted) *	0.145	0.36	0.197	0.21	0.299	0.052
RV/TLC ratio (%) *	−0.128	0.40	−0.068	0.66	**0.500**	**0.001**
Raw (cm H_2_O/L/s) *	−0.118	0.40	−0.074	0.63	0.284	0.058
DLco (% predicted)	0.059	0.70	0.258	0.091	−0.267	0.079
MIP (% predicted)	0.198	0.17	**0.293**	**0.039**	**−0.285**	**0.044**
MEP (% predicted)	0.145	0.31	0.275	0.053	−0.262	0.066
HGS (kgf)	0.251	0.079	0.136	0.35	**−0.335**	**0.017**
QMS (kgf)	**0.373**	**0.007**	**0.291**	**0.040**	**−0.641**	**<0.001**
FACIT-F (points)	0.255	0.074	**0.325**	**0.021**	**−0.340**	**0.015**
6MST (number of steps climbed)	**0.290**	**0.041**	**0.310**	**0.028**	**−0.696**	**<0.001**
6MST (% predicted)	0.183	0.20	0.263	0.065	**−0.615**	**<0.001**

Bold type indicates significant correlations. BBS, Berg Balance Scale; TBS, Tinetti Balance Scale; TUGT, Timed “Up and Go” Test; BMI, body mass index; FVC, forced vital capacity; FEV_1_, forced expiratory volume in 1 s; FEF_25–75%_, forced expiratory flow during the middle half of the FVC maneuver; TLC, total lung capacity; RV, residual volume; Raw, airway resistance; DLco, diffusion capacity of the lungs for carbon monoxide; MIP, maximum inspiratory pressure; MEP, maximum expiratory pressure; HGS, handgrip strength; QMS, quadricep muscle strength; FACIT-F, Functional Assessment of Chronic Illness Therapy-Fatigue; 6MST, six-minute step test. * *n* = 45.

## Data Availability

Data is contained within the article.

## Abbreviations

The following abbreviations are used in this manuscript:

PTLD	Post-tuberculosis lung disease
PTB	Pulmonary tuberculosis
TB	Tuberculosis
HGS	Handgrip strength
QMS	Quadricep muscle strength
DLco	Diffusion capacity of the lungs for carbon monoxide
FCE	Functional capacity during exertion
iwPTLD	Individuals with post-tuberculosis lung disease
BC	Balance control
CNS	Central nervous system
CNSTB	Central nervous system involvement by tuberculosis
COPD	Chronic obstructive pulmonary disease
BBS	Berg Balance Scale
TBS	Tinetti Balance Scale
TUGT	Timed “Up and Go” Test
FACIT-F	Functional Assessment of Chronic Illness Therapy-Fatigue
6MST	Six-minute step test
MIP	Maximum inspiratory pressure
RV	Residual volume
TLC	Total lung capacity
SPH	Static pulmonary hyperinflation
6MWT	6 min walk test
